# Multivariate Analysis of Attitudes, Knowledge and Use of ICT in Students Involved in Virtual Research Seedbeds

**DOI:** 10.3390/ejihpe11010004

**Published:** 2021-01-10

**Authors:** Magda Alejandra Martinez-Daza, Alfredo Guzmán Rincón, Jader Alexis Castaño Rico, Nuria Segovia-García, Harvey Yesid Montilla Buitrago

**Affiliations:** 1 School of Economic and Administrative Sciences, Corporación Universitaria de Asturias, 110221 Bogotá, Colombia; jader.castano@asturias.edu.co (J.A.C.R.); tecnologia.ns@iep.edu.es (N.S.-G.); 2 Graphic Design Faculty, Universidad Santo Tomás, 110231 Bogotá, Colombia; harveymontilla@usantotomas.edu.co

**Keywords:** ICT, attitudes, knowledge, use, research seedbed, formative research, students

## Abstract

The incorporation of information and communication technologies (ICTs) in higher education has been carried out in a transversal manner within the curriculum, and the processes of formative research in both face-to-face and virtual programmes are not an exception to this process. In this context, it is recognised that students’ perceptions of the inclusion of technologies in the classroom can influence their teaching and learning process; however, they have not been widely addressed in multiple settings including research seedbeds. Thus, this paper aims to identify such perceptions represented in the attitudes, knowledge and uses of ICTs in students ascribed to the research seedbed in a virtual business administration programme of an Institution of Higher Education located in Colombia. For its fulfillment, the ACUTIC scale was applied to a sample of 65 students in order to identify these perceptions through a hierarchical cluster analysis, a single factor analysis of variance (ANOVA) test, a post hoc Tukey method and a factor analysis. The main result is that attitudes, knowledge and use of ICTs are varied and they can be represented in three clusters. In general, the attitude towards the incorporation of technologies in the research seedbed is positive; however, there is a gap in terms of knowledge and use, especially of those tools oriented to the disciplinary field and research.

## 1. Introduction

Institutions of higher education (IHE) in Colombia, under the regulatory framework of Decree 1330 of 2019, are responsible for incorporating formative research in the curriculum of undergraduate programs (or the degree as it is known in other Latin American countries), in order to provide transformative responses to local, regional and global problems, and investigate the social and environmental reality, among other things, by using knowledge as a tool for development [1]. In this sense, for the undergraduate level, students are expected to develop analytical and critical thinking by the end of their training process, which will allow them to provide solutions to the problems of the productive sector using research methods and information and communication technologies (ICTs). This combination allows the development of both research and digital skills which are essential in the context of future professional performance [2]. Thus, those programmes taught in the virtual mode are not the exception, and IHEs have sought to implement formative research through different pedagogical strategies such as degree work and research seedbeds in these programmes.

The latter are conceived as an autonomous space for research training in which teachers and students come together to develop and carry out research projects. In the specific case of undergraduate programmes in the virtual mode, plans for actions are mediated by virtual learning environments, which include resources and tools for the development and strengthening of competencies in research and the use of ICTs [3]. These resources and tools, within the framework of the research seedbed, strengthen and autonomous and collaborative work which promotes the virtual integration through activities for teaching and learning concepts related to research methodology, information gathering, report writing, research products, knowledge and the use of programmes related to the research process, as well as web tools, among others [4,5]

Taking into consideration the nature of the seedbed in e-learning programmes, ICTs are the cornerstone of the teaching and learning process of research as a teaching strategy [6], it is necessary to recognise that the teaching and learning process of research is influenced, either positively or negatively, by the students’ attitudes, knowledge and uses of ICTs [7,8,9,10]. In this sense, the literature reports that ICTs improve the educational experience [11,12,13,14,15,16], increase student academic performance [17], and strengthen the teacher–student relationship [18,19]. However, it is necessary to clarify that this research has been focused on the scenarios other than the process of formative research that takes place in research seedbeds. In this context, studies carried out regarding the attitudes, knowledge and use of ICTs by students in relation to this educational process are limited. Nonetheless, those that do exist report that the apprentices with little self-sufficiency in research have a low level of knowledge of the tools and programmes that are fundamental for carrying out research activities, in addition to having a negative attitude towards the processes that take place in the seedbed [20].

Therefore, there is a gap in terms of analysing perceptions related to attitudes, knowledge and uses of ICTs in formative research processes, specifically in research seedbeds. Hence, assessing attitudes towards ICTs, knowledge of ICTs among seedbed students and the uses of ICTs in research processes contribute to strengthening this type of pedagogical strategy [21].

Thus, this paper aims to identify the attitudes, knowledge and uses of ICTs among students enrolled in the research seedbed of a virtual business administration programme at an IHE located in Colombia. It is divided into five main sections: the first provides contextualisation of the use of ICTs in virtual mode programmes in Colombia and the conceptualisation of attitudes, knowledge and uses of ICTs, as well as the progress made in previous research; the second explains the methodology used in the development of this analysis; the third presents the results in accordance with the defined methodology; the fourth presents the discussion; and the fifth presents the conclusions.

### 1.1. Contextualisation of the Use of Information and Communication Technologies (ICTs) in Virtual Mode Programmes in Colombia

In Colombia, the IHEs with virtual programmes are autonomous regarding the incorporation of ICTs in their training programmes, however, they must be in line with the social and economic realities of the students, thus the tools used have to allow the teaching and learning process to take place on an equal footing with the programme being studied [1]. In this sense, the Ministry of National Education recognizes that, although the programmes in this modality are offered nationwide, one of the main challenges they face is to comply with this condition of equality, due to the digital division in the country. Therefore, according to the data from the Ministry of Information and Communication Technologies [22], there is a marked gap in terms of access to internet-based technologies where, by 2019, only 7 million people (14% of the national population) had access to this service on a fixed basis, 4. 1 million in cable access, 1.6 million in xDSL, 1 million in fibre optics and 300,000 in other technologies. On the other hand, the average connection speed was 14.5 Mbps, although in the most rural departments (Vaupés, Guainía, Vichada, Amazonía and San Andrés) this speed was not higher than 2 Mbps. In the case of mobile internet access, 1.3 million users were connected via a 2G network, 8.2 million via a 3G network and 21.3 million via a 4G network. In addition, there are also difficulties in accessing cutting-edge technology in many areas, such as mobile devices and laptops, among others.

Faced with this disruptive panorama, the IHEs with virtual programmes have opted to develop virtual learning environments based on technologies that allow them to overcome the digital division in terms of both, access to the internet and the technologies mediated by it, in order to fulfil the condition of equality in the teaching and learning process described above. In this way, technologies, resources and teaching materials designed for this reality have been incorporated; however, they limit, to a certain extent, the implementation of resources such as the use of mobile applications, 3.0 and 4.0 technologies, among others.

In the case of virtual training programmes in Colombia, various technologies have been implemented in their virtual learning environments. Based on their benefits and barriers, they seek to develop both disciplinary and transversal competences, the latter including digital competences. From the students’ perspective, these skills are understood as the computer skills required by professionals to perform their professional and social roles [23].

Thus, through the development of pedagogical strategies in accordance with the students, the IHEs expect them to know and use programmes on: office automation, information search engines, communication systems, databases and digital libraries, 2.0 tools, social networks for professional purposes, programmes for the design and editing of images and videos, learning and teaching platforms, specialised software for the analysis of both qualitative and quantitative data, for the relationship and social link with academic and professional networks, the creation of portfolios, educational material, blogs, as well as systems for the creation of academic material, etc.

It should be noted that in the case of the research seedbeds, it is expected that ICTs will be incorporated into their teaching strategies, both in the virtual learning environment and in those that merit the development of research projects, respecting in all cases the principle of equality in education [2].

### 1.2. Attitudes, Knowledge and Uses of ICTs

The incorporation of ICTs in higher education has changed the traditional paradigms related to the teaching and learning process positioning them as one of the main tools for: transmitting knowledge, generating interaction between teacher and student, expanding educational coverage, among others. In this context, it is recognized that their integration into undergraduate academic programmes, especially the virtual ones, is transversal to the curriculum, which implies research processes as well. Taking into consideration the previous idea, the literature shows that students’ perceptions are different in terms of their implementation, which generates different attitudes, different levels of knowledge and applicability, represented in their uses.

Thus, when referring to attitudes towards ICTs, these are understood as the observable information, either in a qualitative or quantitative way, behind the cognitive, behavioural and affective responses of students in the incorporation of technologies in the classroom [24,25,26]. Following the latter, the cognitive response refers to students’ beliefs about ICTs [27,28]; affective response refers to students’ feelings about using ICTs (e.g., joy, fear, anxiety, etc.) [26,28,29]; and behavioural response refers to the reinforcement or change of students’ behaviour in relation to the technologies [27]. In this way, the attitude assumes a predisposition of the student to be favourable or unfavourable to the incorporation and use of ICT in their educational process [30].

As far as this perception is concerned, the literature shows that students generally have a positive attitude towards the incorporation of ICT in the classroom, since they consider that the latter has the capacity to make the training process dynamic in its different elements [31,32]. In this sense, it is highlighted by students that the assessments made using technological tools allow for rapid feedback, especially assessments. Furthermore, they consider that these decrease the bias given by teachers’ judgements or values [33,34,35].

On the other hand, technologies applied to the classroom allow self-learning. Students can explore other types of teaching resources which include software, videos, readings, among others [35,36,37]. In addition, the studies refer to the fact that the use of multimedia content allows the integration of some concepts that have already been explained by the teacher improving in that way the development of both disciplinary and transversal competences [37,38].

In the necessity of facing the knowledge and use of ICTs, the analysis is now usually carried out jointly, since the greater the knowledge of a technological tool, the greater the probability of its use by the student. In the case of ICT knowledge, this refers to the skills learned about specific technologies beforehand, while their use gives an account of the applicability of these devices. In this sense, it is evident that ICTs as tools used by students, within the framework of the subjects, facilitate the development and strengthening of competences, make knowledge more accessible and concepts more appropriate [2,39,40]. Similarly, their frequent use allows them to structure new knowledge, based on access, selection, organisation and interpretation of more complex information [28,29,40].

It is also evident from the bibliographic study that the students with a greater knowledge of computer tools related to their field of training tend to give more efficient and creative solutions to problems of a disciplinary nature [41,42,43], as well as focusing on more complex or higher-level tasks, rather than those of an operational or manual nature [44]. Additionally, the use of ICTs, especially those used for data analysis, enhance the development of critical thinking. Thus, the study carried out by McMahon [45,46] showed statistically significant correlations between the use of technological tools and the development of this thought process.

Finally, as regards the integration of ICT in the classroom, it is recognised that students may present certain difficulties associated with the use of ICTs. It has also been identified that the mediation of technologies in certain activities of the teaching and learning process can generate anxiety, especially in the case of assessments [47,48], which affects the attitude to technologies. For that reason, Whelan [49] identified that the advanced use of certain technological tools in the classroom, with which the student is not familiar, generates a situation of uncertainty, and, consequently, an effect on their cognitive processes.

In summary, the previous research related to the attitudes, knowledge and use of ICT by students shows that the integration of technologies in the classroom, in higher education, can be positive or negative; and, for the specific case of aspects related to research processes and more specifically to seedbeds, this type of perception has not been explored in depth.

Based on both the context given in the introduction and the previous literature review, the following research question was addressed: what are the characteristics that students enrolled in the research seedbed of a virtual business administration programme at an IHE located in Colombia possess in terms of attitudes, knowledge and uses of ICTs?

## 2. Materials and Methods 

In order to fulfil the proposed objective, this study is quantitative, exploratory and transversal, using the hierarchical clustering statistical technique. This allows a group of individuals to be subdivided by means of common characteristics [50]. This technique analyses n observed variables with d descriptors, for which the use of discrete variables such as the Likert scale is appropriate. In this sense, this type of analysis is part of the automatic or unsupervised multivariate techniques, which is recommended when the way in which individuals are grouped together is unknown due to the context in which the study is carried out or due to the lack of literature on these groupings [50]. Having said this, hierarchical clustering allows individuals to be characterised on the basis of observable variables and, in the case of education, its use has been varied, focusing on learning styles, attitudes and the development of competencies, among others.

In this context, the study was conducted with a sample of 65 students from a research seedbed in a virtual business administration programme at an IHE located in Colombia. This sample corresponded to the total number of students who participated in the pedagogical strategy in the period 2018 to 2020 (first semester). The students were asked to complete the “Scale for the study of attitude, knowledge and use of ICTs” (ACUTIC by its Spanish acronym) [51], which is a self-report questionnaire that evaluates attitudes, knowledge and uses of ICTs in higher education contexts. It consists of 31 items on a Likert scale as shown in Table 1, Table 2 and Table 3. Comparing the use of this scale in previous studies, Gámez et al. [52], Lopéz et al. [53], Casillas et al. [54] and Figueroa et al. [55] stand out. It is important to recognise that this self-reporting scale exemplifies some software tools which have been used since the 1990s, and their validity lies in their updating and incorporation into virtual learning environments, especially in the promotion and development of competences formative research, as well as their easy adaptability to these environments. On the other hand, the present instrument exemplifies some technological tools, which may not be in force at the time of the application of the scale. However, the purpose of these examples in the instrument is to relate the statement it assesses to some context close to the student, since they may not have access to more recent tools, and therefore, this resource is used to find out the perception of attitudes, use and knowledge of ICT.

In order to determine the clusters, the items that in the factor analysis developed by the authors of the instrument [51] had values lower than 0.5 were eliminated, and were not included: Con_4, Con_10, Usage _3, Usage _4 and Usage _7. After this, the hierarchical cluster analysis was carried out using Wald’s method with Euclidean distance squared and without value transformation. To determine the difference between them, for each one of the variables that the ACUTIC scale evaluates, an analysis of variance (ANOVA) of a factor was developed followed by Tukey’s post hoc statistician. Both the single-factor ANOVA and Tukey’s post-hoc test was considered statistically significant when p was lower than 0.05, identifying in that way the difference between the clusters and the items of the instrument. As regards the use of the ANOVA statistic, it allows the comparison of H0 where the averages of the clusters or conglomerates are the same, so its use is limited when there are at least k independent groups greater than two. In the case of the Tukey post hoc test it is usually used to determine differences between independent groups, where k is greater than three, it is usually after the use of the ANOVA analysis of a single factor, evidencing punctual differences between the variables under study from the means of the variables in the groups.

In addition, an exploratory factor analysis was carried out on the variables using the main method, with the extraction of three factors (corresponding to those evaluated by the ACUTIC scale) to identify the relationship of the clusters with these factors, in addition to analysing specific characteristics of the students. A 95% reliability interval was defined for all the statisticians.

## 3. Results

**Figure 1:**
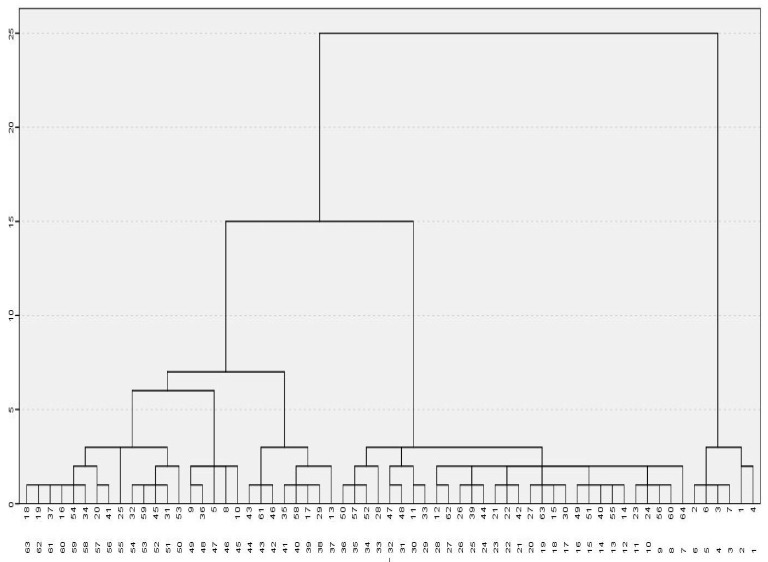
Dendrogram for cluster determination.

Regarding the data collected in the sample, 98.4% were valid for the analysis of the hierarchical cluster, only 1.6% was erased. Therefore, the new sample under study was made up of 64 students. Figure 1 shows the three clusters that were formed by cutting the distance in the scale 10. Hence, the first one was composed by 6 cases equivalent to 9.5% of the sample (2, 6, 3, 7, 1 y 4); the second, 27 cases (42.29%) (18,19, 37, 16, 54, 34, 20, 41, 25, 32, 59, 45, 31, 53, 9, 36, 5, 8, 10, 43, 61, 46, 35, 58, 17, 29 y 13); and the third 30 cases (46.9%) (50, 57, 52, 28, 47, 48, 11, 33, 12, 62, 26, 30, 44, 21, 22, 42, 27, 63, 15, 30, 49, 51, 40 55, 14, 23, 24, 56, 60 y 64).

**Figure 2:**
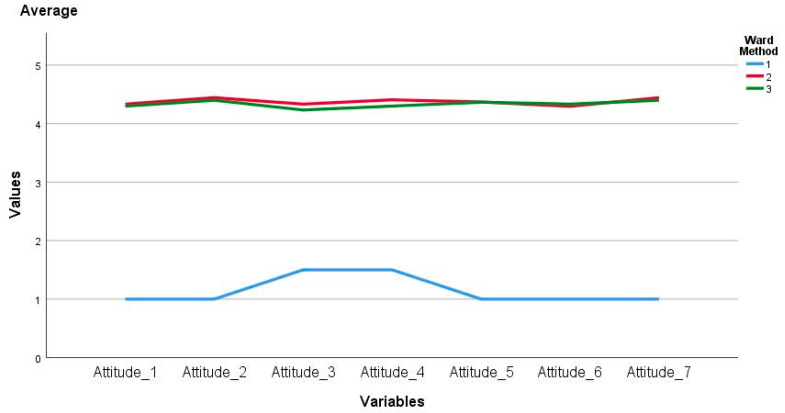
Average attitude factor towards ICTs by cluster.

In the case of the variables that made up the factor called attitudes towards ICTs, statistically significant differences were evidence through the ANOVA analysis in the items of Attitude_1 (F_(2, 60)_ = 27.85, *p* = 0.00), Attitude_2 (F_(2, 60)_ = 31.78, *p* = 0.00), Attitude_3 (F_(2, 60)_ = 21.05, *p* = 0.00),Attitude_4 (F_(2, 60)_ = 22.64, *p* = 0.00), Attitude_5 (F_(2, 60)_ = 30.79, *p* = 0.00), Attitude_6 (F_(2, 60)_ = 29.85, *p* = 0.00) and Attitude_7 (F_(2, 60)_ = 31.78, *p* = 0.00). As a result, Tukey ‘s post-hoc test revealed that such differences occur between cluster one and cluster two and three averages. Thus, for the case of Attitude_1 the difference was −3.33 ± 0.67 with *p* = 0.00 and −3.33 ± 0,63 with *p* = 0.00 respectively; Attitude_2 was −3.44 ± 0.68 with *p* = 0.00 and −3.40 ± 0.65 with *p* = 0.00; Attitude_3 was −2.83 ± 0.62 with *p* = 0.00 and −2.73 ± 0.58 with *p* = 0.00; Attitude_4 was −2.90 ± 0.31 with *p* = 0.00 and −2.80 ± 0.28 with *p* = 0.00; Attitude_5 was −0.37 ± 0.36 with *p* = 0.00 and −3.36 ± 0.33 with *p* = 0.00; Attitude_6 was −3.29 ± 0.24 with *p* = 0.00 and −3.33 ± 0.24 with *p* = 0.00; finally, in the case of Attitude_7 was −3.44 ± 0.31 with *p* = 0.00 and −3.40 ± 0.31 with *p* = 0.00. In the case of clusters two and three no statistically significant differences were established. The above is summarized in Figure 2.

**Figure 3:**
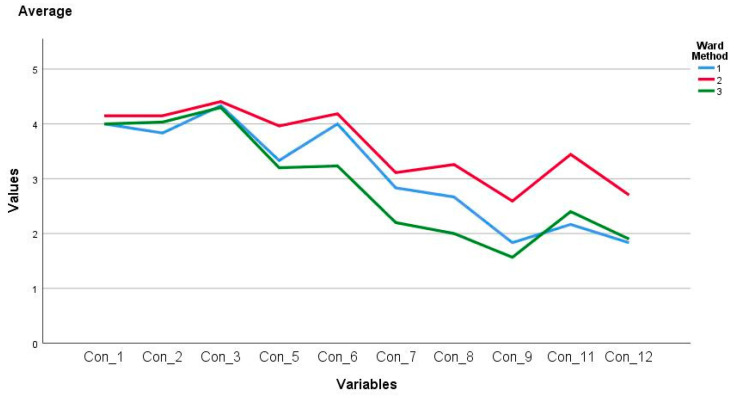
Medium factor ICT knowledge by conglomerate.

Regarding the factor called ICT knowledge, it was identified that there are statistically significant differences between the items Con_5 (F _(2, 60)_ = 10.66, *p* = 0.00), Con_6 (F_(2, 60)_ = 13.67, *p* = 0.00), Con_7 (F_(2, 60)_ = 5.78, *p* = 0.00), Con_8 (F_(2, 60)_ = 11.28, *p* = 0.00), Con_9 (F_(2, 60)_ = 7.60, *p* = 0.00), Con_11(F_(2, 60)_ = 12.91, *p* = 0.00) and Con_12 (F_(2, 60)_ = 4.09 *p* = 0.02); therefore, at least one of the clusters presented divergent averages in some of them. Under this scenario, it was determined that for Con_5 the difference is between cluster two and three with 0.76 ± 0.17 and *p* = 0.00; Con_6 between cluster one and three with 0.76 ± 0.31 and *p* = 0.01, and between cluster two and three with 0.95 ± 0.18 and *p* = 0.00; Con_7, Con_8, Con_9 and Con_ 12 between cluster two and three with 0.91 ± 0.27 and *p* = 0.00, 1.25 ± 0.25 and *p* = 0.00, 1.02 ± 0.22 and *p* = 0.00 and 0.80 ± 0.29 and *p* = 0.02 respectively; and, Con_11 and cluster two and three with −1.27 ± 0.38 and *p* = 0.00 and cluster two and three with 0.80 ± 0.24 with *p* = 0.02. These differences are presented in Figure 3.

**Figure 4:**
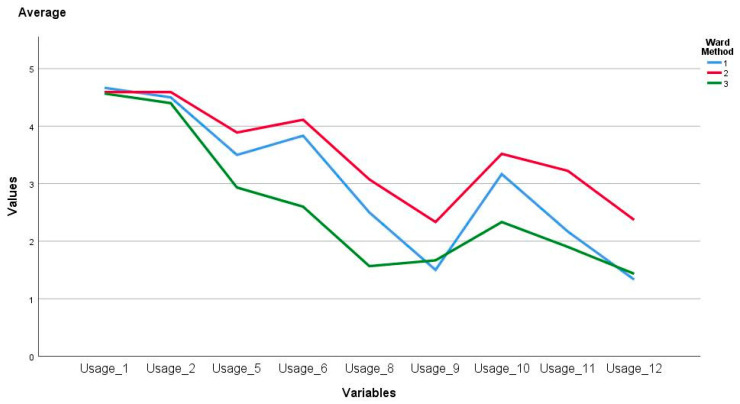
Average use of ICT factor by cluster.

In relation to the variables that evaluate the use of ICT, significant differences were evident between at least one of the clusters for the following items Usage_5 (F_(2, 60)_ = 10.29, *p* = 0.00), Usage_6 (F_(2, 60)_ = 18.59, *p* = 0.00), Usage_8 (F_(2, 60)_ = 20.04, *p* = 0.00), Usage_9(F_(2, 60)_ = 4.53, *p* = 0.01), Usage_10 (F_(2, 60)_ = 13.80, *p* = 0.00), Usage_11 (F_(2, 60)_ = 19.06, *p* = 0.00) and Usage_12 (F_(2, 60)_ = 9.82, *p* = 0.00). Thus, in Tukey’s post-hoc test it was identified that for Usage_5the difference was between cluster two and three with 0.95 ± 0.25 and *p* = 0.00; Usage_6 between one and three with 1.23 ± 0.42 and *p* = 0.01, also, between two and three with 1.50 ± 0.23 and *p* = 0.00; Usage_8, Usage_9 and Usage_10 between cluster two and three with 1.50 ± 0.23 and *p* = 0.00, 0.66 ± 0.24 and *p* = 0.02 and 1.18 ± 0.22 and *p* = 0.00 respectively; Usage_11 and Usage_12 between cluster one and two with −1.05 ± 0.36 y *p* = 0.01 y −1.03 ± 0.38 and *p* = 0.02, finally, for these same items, differences were also related between cluster two and three with 1.32 ± 0.21 and *p* = 0.00 and −0.97 ± 0.22 and *p* = 0.00. They are represented in Figure 4.

In summary, through the ANOVA analysis of a single factor and the Tukey post hoc statistician, it became evident that the students associated with cluster one were characterised by a negative attitude towards ICTs, since they considered that they do not encourage the teaching and learning process, do not improve the quality of this process, are not essential both for their development and that of the class, do not allow the achievement of skills, and do not provide flexibility in space–time for communication with teachers and peers. On the other hand, this conglomerate was self-perceived as having high knowledge in the handling of social networks and low knowledge in the creation of multimedia material in relation to the teaching and learning process. In the case of cluster three, they presented high positive attitudes towards ICT, but with the lowest levels in terms of knowledge and use of these tools. This highlighted their low self-perception with regard to their knowledge of the software used in the research (e.g., SPSS and AtlasTi), a situation which was reflected in their use of these, a similar situation being presented with regard to teaching and learning platforms such as Learning Management Systems. Finally, in cluster two, all the students were grouped together that they consider having positive attitudes, high knowledge and frequent use of ICT.

Regarding the factorial analysis, the Kaiser–Meyer–Olkin (KMO) statistic was 0.74, so the variables were partially and totally strongly correlated. In addition, the Bartlett sphericity test (BTS) had a Chi-square approximation of 1131.92 with p of 0.00, so the variables under study were explained by the factors extracted. Thus, as shown in Table 4, the three factors of the instrument explain 56.80% of variation.

**Figure 5:**
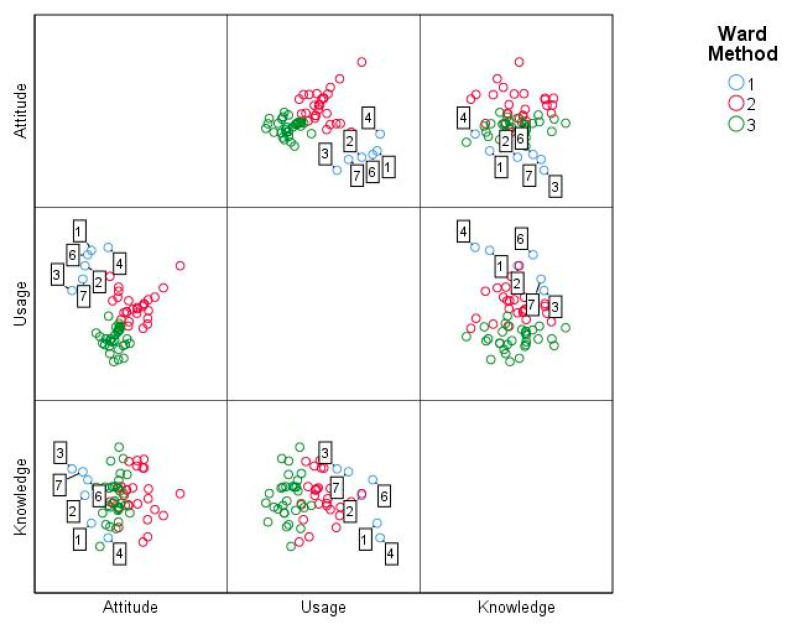
Cluster cases one per factor.

From the three factors, it was determined that for the case of cluster one (see Figure 5) in terms of the use and knowledge of ICT, subject number three had similar characteristics to the subjects of cluster two. In the case of attitude and knowledge of ICT, subject four had similar characteristics to cluster three, and in addition, subject three gave a negative assessment of the implementation of ICT but stated the fact of having extensive knowledge of them. Finally, with regards to the attitude and use of ICTs, no particularities were evident in the cases.

**Figure 6:**
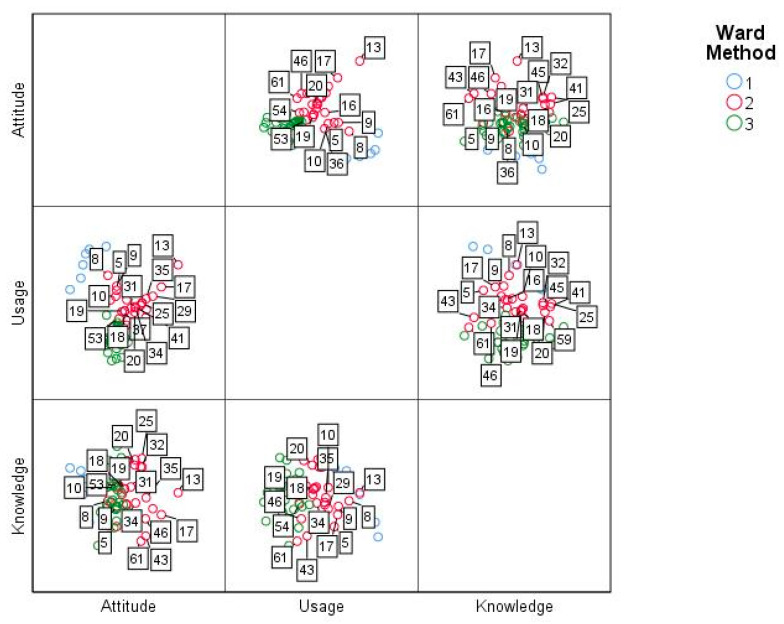
Cluster two cases per factor.

On the other hand, subjects 61 and 43 belonging to cluster two, perceived themselves as non-ICT students with low ICT knowledge; and subject 54, for these two factors, had similar characteristics to subjects belonging to cluster three. In terms of attitude and use of ICT, student 13 stood out, as he tended to make frequent use of the technological tools assessed by the instrument, in addition to having positive attitudes, which were above average for his cluster. In the case of attitudes and knowledge of ICT, it was evident that subjects 59, 20, 41, 25, 32, 31, 58 and 45 considered that they had greater knowledge of these, while students 61 and 43 had positive attitudes in between, but with a low self-perception regarding technological knowledge. Figure 6 shows the relationship of this cluster for each of the factors.

**Figure 7:**
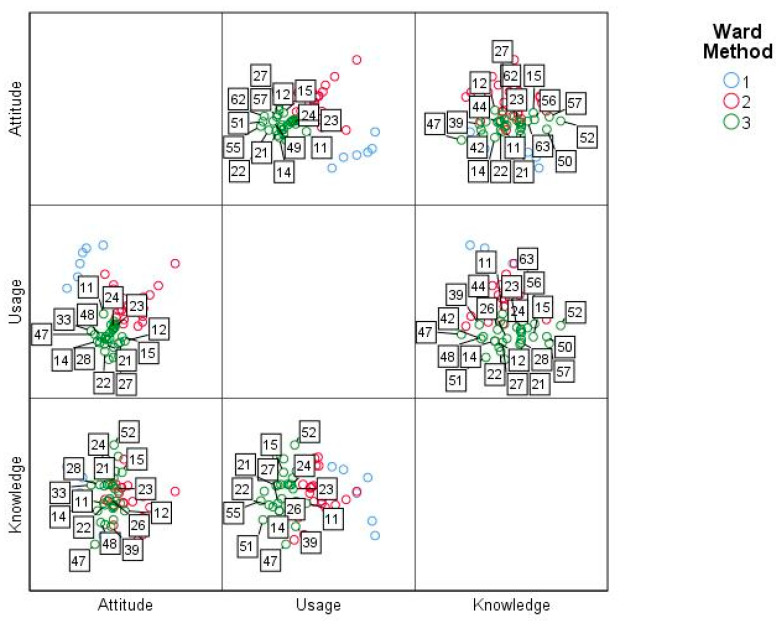
Cluster three cases per factor.

In the case of cluster three, for attitude factors and use of ICT, no particularities were identified in the cases. On the other hand, student 47, who perceived himself as having low knowledge in the use of this type of tool, stood out; while subject 52 considered himself to have knowledge above the average of the cluster. This is represented in Figure 7.

## 4. Discussion

In the area of results, the perceptions of the attitudes, knowledge and use of ICTs by students involved in training research processes, and more specifically in the research seedbed of a virtual programme, showed that these are varied, which is in line with what is expressed in the literature in other training scenarios [11,31,56,57]. Therefore, the majority of students showed a positive attitude towards the use of technological tools in the teaching and learning processes that take place in the seedbeds, and related their use to the improvement of the quality of this process. Taking into consideration the nature of education in the virtual modality, the students considered that it was essential to incorporate various ICTs in the virtual learning environment in which this pedagogical strategy is developed, since they facilitate the development of the class, allow the achievement of competences in this case of research, and provide flexibility in space and time for the development of training activities. This is related to the principle of self-learning that occurs in this study modality, in which the student is the one responsible for managing his or her own learning, so that positive attitudes towards the use of ICT in the classroom reinforce this concept, and, consequently, he or she is capable of developing more complex tasks autonomously [56,58], as well as changing behaviour in the use of ICT [33,59]. On the other hand, it is necessary to highlight that in the case of cluster one, students with low self-perceptions regarding attitudes towards ICT were concentrated. Therefore, they considered that technologies do not encourage the teaching and learning process, and do not represent an improvement in its quality; additionally, they considered that technological tools hinder the development of classes, the achievement of skills, and do not provide flexibility in time.

Regarding the knowledge and use of ICT, the students enrolled in the seedbed generally reported that they know and use desktop and office automation tools, network tools such as search engines (e.g., Google, Yahoo!, Bing), communication systems such as emails or forums, web 2.0 and social networks. The common factor they have is that they are usually used in the students’ daily lives and are not exclusive to the training or research process. Therefore, when they use them frequently, they can explore new functionalities or improve their knowledge of these tools. However, with regard to ICT-related fields, whether disciplinary or research, students in clusters one and three expressed that they do not have high levels of knowledge or use. In line with the above, a representative fraction of students linked to the cluster do not know or use audio, image or video editing programs, tools integrated in virtual learning environments, creation of virtual materials or educational programs. Otherwise, students in cluster two reported extensive knowledge or frequent use of the tools described above. Regarding software specialized in research processes such as Atlas Ti, SPSS, R-Studio, among others, the sample of students believed that they did not have sufficient knowledge or frequent use of them. Based on the above, the results reflect similarities with the findings of Castro and Castro [40], who detailed that the students tend to make frequent use of those technologies with which they are familiar, but in contrast to others, they tend to consider that they do not have the appropriate level of knowledge to make use of them.

However, in view of the limitations of this study, both the results and the scope of the discussion and conclusions must be interpreted based on the size of the sample, so that they cannot be generalized to the student population linked to research seedbeds, business administration programmes or the national education system. On the other hand, given the convergence and accelerated evolution of ICT the scale used presents some examples, which may not be applicable in contexts where state-of-the-art technologies have been incorporated into the teaching and learning processes (e.g., technologies 3.0 and 4.0), so it is necessary to eliminate the biases of development and appropriation of technologies for the analysis of the present, since the programmes in virtual mode in Colombia usually have students with varied profiles ranging from urban to rural, with employment to unemployment, with access to the internet at home or at work to not having it directly, among others [60]. Hence, the incorporation of ICT in the learning classrooms of virtual programmes does not depend only on the intentions of the IHEs or the teacher, but on the technological realities of the country, region, and especially of the student, which in many cases are adverse and the use of robust technologies does not mean a greater experience in the teaching and learning process. Having said that, if state-of-the-art technologies are implemented in the research seedbeds in the virtual modality programmes, it is necessary to analyse the attitudes, knowledge and uses of this type of ICT by the students. Finally, it is necessary to undertake deeper research into the changes of perceptions in front of other variables that can have an impact on these such as rural and urban, access to technology, etc.

## 5. Conclusions

The work presented here fulfilled the objective set out, identifying the attitudes, knowledge and uses of ICTs by students attached to a research seedbed in a virtual business administration programme. In this sense, the findings allow us to conclude that the incorporation of ICTs in training research scenarios has the capacity to improve teaching and learning processes, which is perceived by students as positive while this is reflected in their attitudes. In the case of knowledge and use of technologies, IHEs should design and implement modules to strengthen such knowledge, especially in those of a disciplinary nature or aimed at research, this would allow students to develop their critical thinking, and remove barriers to the use of technologies derived from lack of knowledge of specific tools.

Taking into consideration the latter, the present study enriches the literature regarding such perceptions in specific scenarios, such as research seedbeds, specifically those in the virtual modality. In this sense, research related to attitudes, knowledge and uses of ICTs should be further expanded with the ultimate aim of providing tools to IHEs, and more specifically to teachers on how technologies should be successfully incorporated into the research processes developed by undergraduate students. Finally, the results and findings presented must be interpreted from the limitations of the study, such as the size of the sample or the number of seedbeds analysed, since perceptions may vary by variables such as the student’s social context, age, gender, among others.

## Figures and Tables

**Table 1:** Factor attitudes towards information and communication technologies (ICTs) ^1^.

Id	Item	1	2	3	4	5
Attitude_1	ICT encourages involvement in the teaching and learning processes.					
Attitude_2	Teachers must use ICT to improve the quality of learning processes.					
Attitude_3	It is essential to incorporate ICT into university classrooms.					
Attitude_4	Classes improve as ICTs are incorporated.					
Attitude_5	ICT facilitates the development of classes.					
Attitude_6	ICT enables the achievement of skills.					
Attitude_7	ICT provides flexibility of space and time for communication between members of the educational community.					

^1^ Question: Attitudes towards the use of ICTs. Response options: 1 (totally disagree), 2 (disagree), 3 (indifferent), 4 (agree) and 5 (totally agree). Source [51]. The translation was performed for information purposes, the instrument was applied in Spanish.

**Table 2:** Factor ICT knowledge ^1^.

Id	Item	1	2	3	4	5
Con_1	User tools and basic programs such as Word, Power Point, etc.					
Con_2	Network information search tools such as Google, Yahoo!, Bing, Lycos, etc.					
Con_3	Communication systems. For example, e-mail, forum, chat, videoconference, etc.					
Con_4	Digital libraries and databases.					
Con_5	2.0 Tools. For example, YouTube, Slideshare, Picasa, Flickr, Blogger, Wikispaces, etc.					
Con_6	Spaces for social interaction, such as Tuenti, Facebook, hi5, Pinterest, etc.					
Con_7	Software for image, audio and video editing, such as Photoshop, Pixelmator, Audacity, Power Sound Editor, Windows Movie Maker, iMovie, etc.					
Con_8	Virtual teaching-learning platforms, e.g., Sakai, Moodle, Suma, etc.					
Con_9	Software for data analysis, such as SPSS, Mystat, Nud.ist, Atlas.ti, etc.					
Con_10	Networked educational resources, such as translators, courses, podcast, repositories of learning objects, etc.					
Con_11	Creation of virtual materials and networked resources for teaching and learning such as the e-portfolio, educational website, Wikis, video games, etc.					
Con_12	Author’s educational software. Such as Clic, JClic, Hot Potatoes, NeoBook, etc.					

^1^ Question: Identify the level of knowledge you have as regards the following technologies. Response options: 1 (None), 2 (Low), 3 (Medium), 4 (High) and 5 (Very high) Source [51]. The translation was performed for information purposes, the instrument was applied in Spanish.

**Table 3:** Factor use of ICT ^1^.

Id	Item	1	2	3	4	5
Usage_1	User tools and basic programs such as Word, Power Point, etc.					
Usage_2	Network information search tools such as Google, Yahoo!, Bing, Lycos, etc.					
Usage_3	Communication systems. For example, e-mail, forum, chat, videoconference, etc.					
Usage_4	Digital libraries and databases.					
Usage_5	2.0 Tools. For example, Youtube, Slideshare, Picasa, Flickr, Blogger, Wikispaces, etc.					
Usage_6	Spaces for social interaction, such as Tuenti, Facebook, hi5, Pintest, etc.					
Usage_7	Software for image, audio and video editing, such as Photoshop, Pixelmator, Audacity, Power Sound Editor, Windows Movie Maker, iMovie, etc.					
Usage_8	Virtual teaching-learning platforms, e.g., Sakai, Moodle, Suma, etc.					
Usage_9	Software for data analysis, such as SPSS, Mystat, Nud.ist, Atlas.ti, etc.					
Usage_10	Networked educational resources, such as translators, courses, podcast, repositories of learning objects, etc.					
Usage_11	Creation of virtual materials and networked resources for teaching and learning such as the e-portfolio, educational website, Wikis, video games, etc.					
Usage_12	Author’s educational software. Such as Clic, JClic, Hot Potatoes, NeoBook, etc.					

^1^ Question: Identify the usage that you have of the following technologies. Response options: 1 (None), 2 (Low), 3 (Medium), 4 (High) and 5 (Very high) Source [51]. The translation was performed for information purposes, the instrument was applied in Spanish.

**Table 4:** Total explained variation of ACUTIC factors.

Component	Initial Self-Values			Sums of Loads Squared from the Extraction		
	Total	% Variation	% Accumulated	Total	% Variation	% Accumulated
1	6.87	26.42	26.42	6.87	26.42	26.42
2	4.90	18.86	45.29	4.90	18.86	45.29
3	2.99	11.50	56.80	2.99	11.50	56.80
4	1.68	6.49	63.29			
5	1.30	5.03	68.32			
6	1.12	4.32	72.64			
7	0.99	3.84	76.48			
8	0.78	3.02	79.51			
9	0.74	2.84	82.35			
10	0.58	2.26	84.62			
11	0.54	2.09	86.71			
12	0.49	1.91	88.63			
13	0.46	1.77	90.41			
14	0.38	1.47	91.88			
15	0.36	1.38	93.26			
16	0.31	1,20	94.47			
17	0.26	1.01	95.48			
18	0.24	0.93	96.42			
19	0.19	0.75	97.17			
20	0.18	0.71	97.89			
21	0.13	0.52	98.41			
22	0.11	0.44	98.85			
23	0.10	0.41	99.26			
24	0.08	0.30	99.57			
25	0.06	0.24	99.81			
26	0.04	0.18	100.00			

## Data Availability

The data presented in this study are available on request from the corresponding author. The data are not publicly available due to the current Colombian laws that require the signing of a data transfer contract between the Corporation of Asturias and the applicants.

## References

[1] Ministerio de Educación Nacional de la Republica de Colombia Decreto 1330 del 2019. https://www.mineducacion.gov.co/1759/w3-article-387348.html?_noredirect=1.

[2] Martínez M.A., Guzmán A., Maerín-Caro E. (2020). Semillero de investigación en modalidad virtual: Diseño, implementación y gestión, caso de estudio. Diálogo de Ciencias Sociales, Económicas y Administrativas: Perspectivas Tendencias y Retos.

[3] Corpas-Iguarán E.J. (2010). Virtualización de los semilleros de investigación: Acaso un modelo de continuidad. Rev. Cienc. Salud.

[4] Díaz-López L.M., Ruiz-Claros C., Cuellar-Cuellar K.Y. (2019). Diseño de estrategias para incentivar la participación de los estudiantes del programa Administración de Empresas en los semilleros de investigación de la Universidad de la Amazonía. Rev. Esc. Adm. Neg..

[5] Martínez-Bravo M.C., Sádaba C., Serrano-Puche J. (2018). Desarrollo de competencias digitales en comunidades virtuales: Un análisis de “scolartic”. Prism. Soc..

[6] Martínez M.A., Gázquez J.J. (2019). Aprendizaje y retos para la apropiación e implementación de la investigación en programas de educación virtual: Caso fundación universitaria del área andina. Innovación Docente e Investigación en Ciencias, Ingeniería y Arquitectura.

[7] Pelgrum W.J. (2001). Obstacles to the integration of ICT in education: Results from a worldwide educational assessment. Comput. Educ..

[8] Nabeel A., Shahrir J., Chin Hai L. (2013). Measuring Attitudes toward Computer and Internet Usage among Postgraduate Students in Malaysia. Turk. Online J. Educ. Technol..

[9] Edmunds R., Thorpe M., Conole G. (2012). Student attitudes towards and use of ICT in course study, work and social activity: A technology acceptance model approach: Exploring student perceptions of ICT in three contexts. Br. J. Educ. Technol..

[10] Vroman K.G., Arthanat S., Lysack C. (2015). “Who over 65 is online?” Older adults’ dispositions toward information communication technology. Comput. Hum. Behav..

[11] Sánchez J., Salinas A., Contreras D., Meyer E. (2011). Does the New Digital Generation of Learners Exist? A Qualitative Study. Br. J. Educ. Technol..

[12] Selwyn N. (2007). The use of computer technology in university teaching and learning: A critical perspective. J. Comput. Assist. Learn.

[13] López-Pérez M.V., Pérez-López M.C., Rodríguez-Ariza L. (2011). Blended learning in higher education: Students’ perceptions and their relation to outcomes. Comput. Educ..

[14] Benson S.N.K., Ward C.L. (2013). Teaching with Technology: Using Tpack to Understand Teaching Expertise in Online Higher Education. J. Educ. Comput. Res..

[15] Tømte C., Enochsson A., Buskqvist U., Kårstein A. (2015). Educating online student teachers to master professional digital competence: The TPACK-framework goes online. Comput. Educ..

[16] Amhag L., Hellström L., Stigmar M. (2019). Teacher Educators’ Use of Digital Tools and Needs for Digital Competence in Higher Education. J. Digit. Learn. Teach. Educ..

[17] Waycott J., Bennett S., Kennedy G., Dalgarno B., Gray K. (2010). Digital divides? Student and staff perceptions of information and communication technologies. Comput. Educ..

[18] Slechtova P. (2015). Attitudes of Undergraduate Students to the Use of ICT in Education. Procedia Soc. Behav. Sci..

[19] Acosta-Núñez J.N., Parrales-Poveda M.L., Arcos-Coba A.P. (2017). Aplicación de las herramientas TICs en el proceso enseñanza-aprendizaje. Dominio De Las Cienc..

[20] Criollo M., Romero M., Fontaines-Ruiz T. (2017). Autoeficacia para el aprendizaje de la investigación en estudiantes universitarios. Psicol. Educ..

[21] Pozo M.A., Cruz M.A. (2020). Contenido científico en la formación investigativa a través de las TIC en estudiantes universitarios. e-Cienc. De La Inf..

[22] Ministerio de Tecnologías de la Información y Comunicación (2020). Boletín trimestral de las TIC. https://colombiatic.mintic.gov.co/679/articles-125648_archivo_pdf.pdf.

[23] Levano-Francia L., Sanchez S., Guillén-Aparicio P., Tello-Cabello S., Herrera-Paico N., Collantes-Inga Z. (2019). Digital Competences and Education. Propósitos Represent..

[24] Cheung D. (2009). Developing a Scale to Measure Students’ Attitudes toward Chemistry Lessons. Int. J. Sci. Educ..

[25] Guitart-Aced R. (2002). Las Actitudes en el Centro Escolar.

[26] Huskinson T.L.H., Haddock G. (2004). Individual differences in attitude structure: Variance in the chronic reliance on affective and cognitive information. J. Exp. Soc. Psychol..

[27] Maio G., Haddock G., Maio R. (2004). Theories of Attitude.

[28] Casillas S., Cabezas M., García F.J. (2020). Digital competence of early childhood education teachers: Attitude, knowledge and use of ICT. Eur. J. Teach. Educ..

[29] Chiao C., Chiu C.-H. (2018). The Mediating Effect of ICT Usage on the Relationship between Students’ Socioeconomic Status and Achievement. Asia-Pac. Edu. Res..

[30] Knezek G., Christensen R., Voogt J., Knezek G. (2008). The Importance of Information Technology Attitudes and Competencies in Primary and Secondary Education. International Handbook of Information Technology in Primary and Secondary Education.

[31] Herrador-Alcaide T.C., Hernández-Solís M. (2016). Educación Digital Contable mediante Redes de Innovación: Una Medición de su Impacto. Digit. Educ. Rev..

[32] Chen C.-L., Wu C.-C. (2020). Students’ Behavioral Intention to Use and Achievements in ICT-Integrated Mathematics Remedial Instruction: Case Study of a Calculus Course. Comput. Educ..

[33] Alsadoon H. (2017). Students’ perceptions of E-assessment at Saudi Electronic University. Turk. Online J. Educ. Technol..

[34] Silva R., Rodrigues R., Leal C. (2019). Play it again: How game-based learning improves flow in Accounting and Marketing education. Account. Eduation..

[35] Van Wyk M.M. (2017). Exploring student teachers’ views on e-portfolios as an empowering tool to enhance self-directed learning in an online teacher education course. Aust. J. Teach. Educ..

[36] De la Fuente Sánchez D., Solís M.H., Martos I.P. (2013). El mini video como recurso didáctico en el aprendizaje de materias cuantitativas. RIED.

[37] Johnston J., Killion J., Oomen J. (2005). Student satisfaction in the virtual classroom. Internet J. Allied Health Sci. Pract..

[38] Brecht H.D., Ogilby S.M. (2008). Enabling a comprehensive teaching strategy: Video lectures. J. Inf. Technol. Educ..

[39] Brush T., Glazewski K.D., Hew K.F. (2008). Development of an Instrument to Measure Preservice Teachers’ Technology Skills, Technology Beliefs, and Technology Barriers. Comput. Sch..

[40] Castro J.J., Alemán C.E. (2011). Teachers’ opinion survey on the use of ICT tools to support attendance-based teaching. Comput. Educ..

[41] Ching C., Joyce L.K., Chin-Chung T. (2010). Facilitating Preservice Teachers’ Development of Technological, Pedagogical, and Content Knowledge (TPACK). Educ. Technol. Soc..

[42] Vega M.E., Morales D., Graverán B.A. (2020). Knowledge of the harmful effects of ICT of students of the Latin American School of Medicine. Cuba Salud.

[43] Rajabion L., Wakil K., Badfar A., Naeini S.M., Zareie B. (2019). A New Model for Assessing the Impact of ICT and Digital Knowledge on Students’ Thoughts and Beliefs. J. Eng. Des. Technol..

[44] Levin T., Wadmany R. (2006). Teachers’ Beliefs and Practices in Technology-based Classrooms. J. Res. Technol. Educ..

[45] McMahon G. (2009). Critical Thinking and ICT Integration in a Western Australian Secondary School. Educ. Technol. Soc..

[46] Fernández-Batanero J.M., Cabero J., López E. (2019). Knowledge and Degree of Training of Primary Education Teachers in Relation to ICT Taught to Students with Disabilities. Br. J. Educ. Technol..

[47] Frederick G.R., Schweizer H., Lowe R. (2006). After the In-Service Course. Comput. Sch..

[48] Hossain M.A., Sormunen E. (2019). ICT Skills of Library and Information Science (LIS) Students in Bangladesh. Int. Inf. Libr. Rev..

[49] Whelan R. (2008). Use of ICT in education in the South Pacific: Findings of the Pacific eLearning Observatory. Distance Educ..

[50] Tan P.-N., Steinbach M., Kumar V., Karpatne A. (2019). Introduction to Data Mining.

[51] Mirete A.B., García F.A., Hernández F. (2015). Cuestionario para el estudio de la actitud, el conocimiento y el uso de TIC (ACUTIC) en Educación Superior. Estudio de fiabilidad y validez. Rev. Interuniv. Profr..

[52] Gámez F.D.G., Peña M.P. (2020). Análisis univariante de la competencia digital en educación física: Un estudio empírico (Univariate analysis of digital competence in physical education: An empirical study). Retos.

[53] Lopéz I.P., Hernández L.G.J., Tobón S. (2020). Construcción y validación de un instrumento para evaluar el abordaje de la sociedad del conocimiento en docentes. Apunt. Univ..

[54] Casillas S., Cabezas M., Sanches-Ferrerira M., Teixeira F.L. (2018). Estudio Psicométrico de un cuestionario para medir la competencia digital de estudiantes universitarios (CODIEU). Educ. Knowl. Soc..

[55] Figueroa V., Burgos F., Guerrero M. (2017). Attitude of teachers toward using the computer in schools in Dominican Republic. Pixel-Bit. Rev. Medios Educ..

[56] Teo T., Kabakç I., Ursavaş Ö.F. (2014). Exploring the digital natives among pre-service teachers in Turkey: A cross-cultural validation of the Digital Native Assessment Scale. Interact. Learn. Environ..

[57] Gross B., Garcia I., Escofet A. (2012). Beyond the net generation debate: A comparison of digital learners in face-to-face and virtual universities. IRRODL.

[58] Web M., Cox M. (2004). A review of pedagogy related to information and communications technology. J. Technol. Pedagog. Educ..

[59] Bush T., Saye J. (2009). Strategies for Preparing Preservice Social Studies Teachers to Effectively Integrate Technology: Models and Practices. Contemp. Issues Technol. Teach. Educ..

[60] Orella D., Segovia N., Grillo C.M., Valencia L.I., Coy H.V., Rodríguez B., Pérez-Fuentes M.C. (2019). El perfil del estudiante adulto en educación superior virtual y su influencia en el abandono. Innovación Docente e Investigación en Ciencias de la Educación y Ciencias Sociales.

